# Actinorhizal Signaling Molecules: *Frankia* Root Hair Deforming Factor Shares Properties With NIN Inducing Factor

**DOI:** 10.3389/fpls.2018.01494

**Published:** 2018-10-18

**Authors:** Maimouna Cissoko, Valérie Hocher, Hassen Gherbi, Djamel Gully, Alyssa Carré-Mlouka, Seyni Sane, Sarah Pignoly, Antony Champion, Mariama Ngom, Petar Pujic, Pascale Fournier, Maher Gtari, Erik Swanson, Céline Pesce, Louis S. Tisa, Mame Oureye Sy, Sergio Svistoonoff

**Affiliations:** ^1^Laboratoire Commun de Microbiologie, Institut de Recherche pour le Développement/Institut Sénégalais de Recherches Agricoles/Université Cheikh Anta Diop, Centre de Recherche de Bel Air, Dakar, Senegal; ^2^Laboratoire Mixte International Adaptation des Plantes et Microorganismes Associés Aux Stress Environnementaux, Centre de Recherche de Bel Air, Dakar, Senegal; ^3^Laboratoire Campus de Biotechnologies Végétales, Département de Biologie Végétale, Faculté des Sciences et Techniques, Université Cheikh Anta Diop, Dakar, Senegal; ^4^Laboratoire des Symbioses Tropicales et Méditerranéennes, Institut de Recherche pour le Développement/INRA/CIRAD, Université Montpellier/SupAgro, Montpellier, France; ^5^UMR 7245, Molécules de Communication et Adaptation des Microorganismes, Muséum National d’Histoire Naturelle, Centre National de la Recherche Scientifique, Sorbonne Universités, Paris, France; ^6^Laboratoire de Botanique et de Biodiversité Végétale, Département de Biologie Végétale, Faculté des Sciences et Techniques, Université Cheikh Anta Diop, Dakar, Senegal; ^7^UMR Diversité Adaptation et Développement des Plantes (DIADE), Institut de Recherche pour le Développement, Montpellier, France; ^8^Ecologie Microbienne, UMR 5557 CNRS, Université Lyon 1, Villeurbanne, France; ^9^Institut National des Sciences Appliquées et de Technologie, Université Carthage, Tunis, Tunisia; ^10^Department of Molecular, Cellular, and Biomedical Sciences, University of New Hampshire, Durham, NH, United States

**Keywords:** symbioses, nodulation factors, nodule inception, *Casuarina*, *Alnus*, *Discaria*

## Abstract

Actinorhizal plants are able to establish a symbiotic relationship with *Frankia* bacteria leading to the formation of root nodules. The symbiotic interaction starts with the exchange of symbiotic signals in the soil between the plant and the bacteria. This molecular dialog involves signaling molecules that are responsible for the specific recognition of the plant host and its endosymbiont. Here we studied two factors potentially involved in signaling between *Frankia casuarinae* and its actinorhizal host *Casuarina glauca:* (1) the Root Hair Deforming Factor (CgRHDF) detected using a test based on the characteristic deformation of *C. glauca* root hairs inoculated with *F. casuarinae* and (2) a NIN activating factor (CgNINA) which is able to activate the expression of *CgNIN*, a symbiotic gene expressed during preinfection stages of root hair development. We showed that CgRHDF and CgNINA corresponded to small thermoresistant molecules. Both factors were also hydrophilic and resistant to a chitinase digestion indicating structural differences from rhizobial Nod factors (NFs) or mycorrhizal Myc-LCOs. We also investigated the presence of CgNINA and CgRHDF in 16 *Frankia* strains representative of *Frankia* diversity. High levels of root hair deformation (RHD) and activation of Pro*CgNIN* were detected for *Casuarina*-infective strains from clade Ic and closely related strains from clade Ia unable to nodulate *C. glauca*. Lower levels were present for distantly related strains belonging to clade III. No CgRHDF or CgNINA could be detected for *Frankia coriariae* (Clade II) or for uninfective strains from clade IV.

## Introduction

Legumes and actinorhizal plants form a N_2_-fixing root nodule symbiosis in association with rhizobia and *Frankia* bacteria, respectively (Vessey et al., 2005). The establishment of these beneficial bacterial-plant relationships requires communication between the partners. Rhizobial symbioses have received considerable attention because several legumes are important crop species. However, actinorhizal symbioses, which play an important ecological role (Dawson, 2008), have been less well studied and the molecular dialog between *Frankia* and their host plants is still poorly understood. One reason is that most actinorhizal plants are woody shrubs or trees for which genetic approaches are very difficult (Wall, 2000; Perrine-Walker et al., 2011). In addition, the genetics of the bacterial partner, *Frankia*, is not fully developed and up to now *Frankia* cells remain recalcitrant to stable genetic transformation (Kucho et al., 2009, 2017). Recent progress including the sequencing of several *Frankia* genomes (Normand et al., 2007; Tisa et al., 2016), transcriptomic studies (Alloisio et al., 2010; Benson et al., 2011), proteomic studies (Mastronunzio and Benson, 2010; Ktari et al., 2017) together with functional studies on several actinorhizal species (Svistoonoff et al., 2014) have opened new avenues for identifying components involved in the initial symbiotic dialog between the two partners.

The interaction of rhizobia with model legumes begins with the production and recognition of signal molecules by their respective eukaryotic and prokaryotic symbiotic partners (Oldroyd, 2013). Early events leading to nodule formation involve bacterial penetration into their hosts *via* root hairs. Bacteria elicit the stimulation and reorientation of root hair cell wall growth. This rhizobia-induced tip growth results first in the entrapment of the bacteria within curled root hairs and then in the initiation and development of infection threads (ITs), tubular structures through which bacteria pass on their way down the root hair and into the underlying cortical cell layers (Lhuissier et al., 2001). Ahead of the advancing threads, cells in the inner cortex are induced to dedifferentiate and divide, and a nodule primordium is formed. In the first part of the signal exchange, the plant roots secrete flavonoids that lead to the activation of a set of rhizobial genes (the *nod* genes), which are essential for infection, nodule development and the control of host specificity (Masson-Boivin et al., 2009; Oldroyd, 2013). These genes are responsible for the synthesis of lipo-chito-oligosaccharides (LCOs) called Nod factors (NFs) that signal back to the plant (Oldroyd, 2013). NF biosynthesis is dependent on *nodABC* genes which are present in all rhizobia able to synthetize NFs and strain-specific combinations of other nodulation genes responsible for the addition of various decorations to the core structure. (Masson-Boivin et al., 2009). In model legumes, NFs perception elicits a range of responses including ion fluxes, calcium oscillations, changes in gene expression patterns, and extensive deformation of roots hairs, which has been used as a bioassay to identify the chemical nature of NFs (Lerouge et al., 1990; Oldroyd, 2013).

Much less is known about signaling molecules involved in the actinorhizal symbioses. Canonical *nodABC* genes are not found in the sequenced genomes of 36 *Frankia* strains including *Frankia alni* and *Frankia casuarinae* (Tisa et al., 2016) confirming a previous report showing that *F. alni* DNA will not complement rhizobial *nod* mutants (Cérémonie et al., 1998). Only distant homologs of *nodB* and *nodC* are found in *F. alni* genome. Unlike rhizobial *nod* genes, they are not organized into a cluster together with other symbiotic genes and their expression is not induced under symbiotic conditions (Normand et al., 2007; Alloisio et al., 2010). These findings are consistent with experiments showing that chitin oligomers similar to rhizobial NFs are not be detected in *F. alni* culture supernatant (Cérémonie et al., 1999) suggesting structural differences between the *Frankia* symbiotic signals and rhizobial NFs. Recently, canonical *nodABC* genes have been found in the genome of two uncultured *Frankia* strains: Candidatus *Frankia datiscae* Dg1 and Candidatus *Frankia californicae* Dg2 (Persson et al., 2015; Nguyen et al., 2016), and in one isolated strain, *Frankia* sp. NRRL B-16219 (Ktari et al., 2017). *F. datiscae* Dg1 *nodABC* genes are arranged in two operons which are expressed in *Datisca glomerata* nodules, but their involvement in symbiotic signaling is still not known (Persson et al., 2015).

*Frankia* is able to infect their host root either through intracellular (root hair) or intercellular modes. In the first case, one of the earliest visible plant response to *Frankia* is an extensive deformation of root hairs. This response occurs in actinorhizal plants belonging to the order Fagales (*Betulaceae, Casuarinaceae*) that display a range of relatively advanced features reminiscent of model legumes: a complex root hair infection process involving the formation of ITs and the implication of cortical cell divisions at the initial stages of infection (Svistoonoff et al., 2014). *Frankia* culture supernatants also cause root hair deformation (RHD) and a *Frankia* root hair deforming factor in *Alnus* (AgRHDF) was identified (Prin and Rougier, 1987; Ghelue et al., 1997; Cérémonie et al., 1999; Gabbarini and Wall, 2011). Using RHD as a bioassay, partial purification was achieved. AgRHDF is a relatively small (< 3 kDa), heat stable, hydrophilic molecule that is resistant to a chitinase treatment, but its chemical structure remains unknown (Cérémonie et al., 1999).

In recent years, we have developed complementary bioassays using plant genes that are specifically expressed in response to interaction with a compatible *Frankia*. This approach is particularly well suited for *C. glauca* where transgenic plants containing promoters of symbiotic genes fused to either *GUS* or *GFP* can be generated (Svistoonoff et al., 2010a). Expressed Sequence Tag (EST) libraries of *C. glauca* and *Alnus glutinosa* (Hocher et al., 2006, 2011) provide extensive lists of genes potentially involved in the actinorhizal symbiosis. Among the candidate genes, we identified *CgNIN*, the putative ortholog of legume *NIN* genes, which encodes a transcription factor playing a central role in rhizobial nodulation (Schauser et al., 1999; Marsh et al., 2007; Soyano et al., 2013, 2014; Yoro et al., 2014). In *C. glauca, CgNIN* also has an important role in nodulation particularly at early steps of infection (Clavijo et al., 2015). After contact with either *Frankia* cells or cell-free *Frankia* supernatants, the *CgNIN* promoter is strongly activated at 12 to 48 h (Clavijo et al., 2015). This property was used to establish a new bioassay leading to the partial purification and characterization of a NIN activating factor, called CgNINA. While rhizobial NFs are amphiphilic chitin-based molecules, CgNINA, like AgRHDF, is hydrophilic and resistant to chitinase (Chabaud et al., 2016). However, it is not known to what extent CgNINA is related to factors able to deform root hairs.

Further experiments concerning these *Frankia* symbiotic factors are reported here. We show that *C. glauca* was able to perceive a root hair deforming factor secreted by *F. casuarinae* (CgRHDF) and the properties of CgRHDF were compared to those previously identified with CgNINA. The presence of CgNINA and CgRHDF in strains representative of *Frankia* diversity was investigated.

## Materials and Methods

### Plant Material and Growth Conditions

*Casuarina glauca* seeds (seed lot 15.934, ref.086-5929) were provided by the Australian Tree Seed Centre^1^. *Ochetophila trinervis* (= *Discaria trinervis*) seeds were collected from plants growing in Pampa de Huenuleo (Bariloche, Argentina). *A. glutinosa* seeds were harvested from a tree situated in the left bank of Rhône River in Lyon, France. *C. glauca* and *O. trinervis* seeds were disinfected and germinated in a sterilized substrate for three weeks and transferred into glass tubes filled with a modified Broughton and Dilworth (BD) medium as described previously (Ngom et al., 2016). *A. glutinosa* seeds were washed in distilled sterile water for 30 min before sterilization in 96% ethanol for 30 min followed by 3% solution of calcium hypochlorite for 30 min. Seeds were germinated on 1.5% plant agar for 10 days at 20°C, and transferred in 5 mL tubes containing liquid Fahraeus medium without nitrogen (Fahraeus, 1957). Plants were grown for 6 weeks in growth chamber at 25° C at 75% relative air humidity and 16 h light cycle/day. Transgenic *C. glauca* plants containing a Pro*CgNIN:GFP* construct described previously (Clavijo et al., 2015) were grown in hydroponics in pots containing the modified BD medium and vegetatively propagated as described previously (Svistoonoff et al., 2010b).

### Preparation of Cell-Free Supernatants and Inoculation

The bacterial strains used in this study are listed in **Supplementary Table S1** and were grown for twenty-one days in modified basic propionate (BAP) media described previously (Ngom et al., 2016) according to conditions listed in **Supplementary Table S1**. Bacterial cultures were exposed to plant root exudates (RE) for five days as described previously (Beauchemin et al., 2012; Clavijo et al., 2015; Chabaud et al., 2016). Cell-free supernatant fluids were purified from cultures showing an absorbance of 0.3 at 595 nm. Cultures were collected by centrifugation at 4,000 g for 5 min and the supernatant fluids were filtered through a 0.22 μm filter as described in Chabaud et al., 2016. Unless otherwise indicated, experiments were performed with the supernatant fluids of a *F. casuarinae* culture induced with RE from its host plant *C. glauca* and referred to as FCS for *Frankiacasuarinae* supernatant. FCS were concentrated fifty times (FCS 50X) using an R-210/215 evaporator (BÜCHI Labortechnik AG, Switzerland). Nodulation experiments with the different *Frankia* strains were performed as described previously (Alloisio et al., 2010; Svistoonoff et al., 2010b; Imanishi et al., 2011).

### Characterization of *F. casuarinae* Supernatant Fluids

#### Temperature and pH Sensitivity

To test heat inactivation, FCS 50X was autoclaved at 120°C for 20 min. Cold sensitivity was determined by freezing FCS at -80°C for 1 h. The effects of pH on FCS activity were determined as follows: the initial pH of FCS (6.7) was adjusted to pH 3, 5, 7, 8 or 10 by adding either HCl or KOH solutions. The FCS mixtures that these pH values were incubated for 1h at room temperature and neutralized back to pH 6.7 by adding either HCl or KOH solutions before performing the bioassays described below. Samples that lost CgNINA or CgRHDF activities were sonicated for 30 min using a Branson 2510 sonicator.

#### Size Fractionation

To estimate the size of signaling molecules, the FCS samples were dialyzed as described in Chabaud et al., 2016. Ten mL of FCS 50X were dialyzed for 12h at 4°C against 5 L of ultrapure water with stirring and using either a 100–500, 500–1000 or 3500–5000 Da cutoff membrane (Float-A-Lyzer G2 dialysis devices, Spectrum Laboratories, CA, United States). The dialyzed solutions were tested using the CgRHD and CgNINA bioassays described below. The size of active compounds was also estimated using centrifugal filters with cutoffs of 30, 10, and 3 kDa (Amicon Ultra-4 centrifugal filters; Merck-Millipore, Cork, Ireland). Four mL of 50X FCS were loaded on a 30 kDa cell which was spun at 4,000 g for 20 min. The filtrate recovered from the 30 kDa filtration was treated similarly using a 10 kDa cell, and the resulting 10 kDa filtrate was added to a 3 kDa Cell and spun.

#### Phase Extraction and Sensitivity to Chitinase

Two sequential butanol extractions were performed on FCS 50X with a ratio 1-butanol / water (1:3; v/v) described previously (Chabaud et al., 2016). The butanol phase was evaporated at 80°C under a nitrogen flow and the residue was dissolved in 20% acetonitrile as described in (Chabaud et al., 2016). Chitinase digestions were performed on the aqueous phase extract as described previously (Chabaud et al., 2016). Chitinase activity was assessed using a colorimetric method to estimate the amount of p-nitrophenol (p-NP) released from a reaction mixture containing the substrate p-nitrophenyl N-acetyl glucosaminide (p-NP-NAG) (Parham and Deng, 2000). A solution containing 1 mg mL^-1^ of *Streptomyces griseus* chitinase (C6137; Sigma-Aldrich) in a 50 mM phosphate buffer pH 6.0 was prepared. A portion of this solution (100 μl) was mixed with 50 μl of 5X FCS aqueous extract, 100 μl of p-NP-NAG solution at10 mM and 250 μl of acetate buffer (pH 5.5 0,1 M). The reaction was incubated at 37°C for 1 h under stirring and was terminated by the addition of 250 μl of CaCl_2_ at 0.5 M and 1000 μl of NaOH at 0.5 M. The amount of p-NP released was evaluated by measuring the absorbance at 400 nm with a spectrophotometer. Enzyme activity was expressed in μg of liberated p-NP per hour of incubation. Control reactions were performed without p-NP-NAG, without chitinase and without the aqueous FCS extract.

### Root Hair Deformation and NINA Bioassays in *Casuarina glauca*

Unless otherwise indicated, CgRHD and CgNINA bioassays were performed on aliquots of the same solution and the amount needed to achieve a final concentration equivalent a 10^-2^ dilution of raw (no diluted) *Frankia* culture supernatant was added to the nitrogen-free BD medium. All experiments were performed on at least 4 plants. At least two independent experiments were carried out for each tested solution.

**FIGURE 1:**
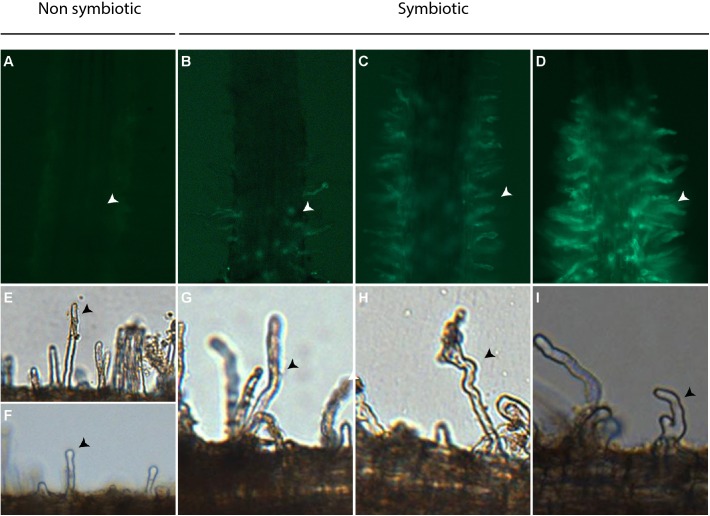
Bioassays used to quantify the activation of Pro*CgNIN* and root hair deformation in *C. glauca.* **(A–D)** Representative images showing levels of GFP fluorescence in root hairs of transgenic Pro*CgNIN:GFP C. glauca* plants used for the CgNINA bioassay. Arrowheads indicate root hairs. **(A)** no signal, level 0; **(B)** weak signal, level 1; **(C)** medium signal, level 2; **(D)** strong signal, level 3. **(E–I)** representative images showing levels of root hair deformation in *C. glauca* used in the CgRHDF bioassay. **(E)** no deformation, level 0a; **(F)** tip swelling, level 0b; **(G)** one change in growth direction, level 1; **(H)** more than one change in growth direction but no bifurcation;, level 2; **(I)** one or more bifurcations, level 3. Arrowheads indicate deformed root hairs.

The deformation of *C. glauca* root hairs (CgRHD) was evaluated using 3 week-old non transgenic plants grown in glass tubes exposed to nitrogen starvation for one week as described previously (Ngom et al., 2016). Treatments were performed by replacing the medium with fresh nitrogen-free BD medium containing the assayed solution. Deformation of root hairs situated on small lateral roots was scored as described by Clavijo et al. (2015) using micrographs taken with a BX50F microscope (Olympus) equipped with a Micro Publisher 3.3 RTV (Qimaging) digital camera. A blind evaluation of each micrograph was performed to determine the deformation level of observed root hairs using the following scale based on Clavijo et al. (2015): 0a: no deformation; 0b: straight root hair with tip swelling; 1 weak deformation, only one change in growth direction; 2: intermediate deformation, more than one change in growth direction but no bifurcation; and 3: strong deformation: one or more bifurcations (**Figure 1**). Deformation levels 0a and 0b were considered non-symbiotic. For each experiment at least four plants were analyzed per treatment and 6 small lateral roots were analyzed per plant. The total number of root hairs scored for each level of symbiotic deformation was used for the statistical analyzes described below. Each experiment was repeated four times independently.

For each treatment the percentage of symbiotic deformation (%SyD) defined as the proportion of root hairs showing a symbiotic response was calculated and used to determine a deformation index using the following scale: level 1: SyD < 15%; level 2: 16% < SyD < 25%; level 3: 26% < SyD < 40%; level 4: SyD > 41%.

The activation of *ProCgNIN* in response to tested solutions was evaluated using transgenic Pro*CgNIN:GFP* plants that were grown in hydroponics deprived of nitrogen for one week as previously described (Clavijo et al., 2015; Chabaud et al., 2016). After 24 h of contact with tested solutions, GFP fluorescence was monitored in the short lateral root hairs using an AZ100 macroscope (Nikon) equipped with a 5X objective, a GFP filter (Excitation filter 470 nm ± 40 nm; Barrier filter 535 nm ± 50 nm; Nikon) and a digital camera Sight D5 RI1 (Nikon). For each observation, a blind evaluation of GFP fluorescence levels was performed using the following scale: 0: no detectable fluorescence; 1: weak fluorescence; 2: intermediate fluorescence; and 3: strong fluorescence (**Figure 1**). For each experiment the number of plants with a given fluorescence level was used for the statistical analysis described below. Each experiment was repeated at least four times independently.

### Root Hair Deformation Bioassay in *A. glutinosa*

The deformation of *A. glutinosa* root hairs (AgRHD) was evaluated using 7 week-old plants that had at least four well developed secondary roots. Biological tests were performed at 10^-2^ final dilutions of *Frankia* culture supernatant fluids on plants growing in 5 ml Fahraeus media without nitrogen. Evaluation of deformation was done in the region located about 1.5 cm from the root tip and five levels of RHD were recorded for each observed root: 0a: no deformation; 0b: swelling; 1: branching; 2: branching and partial deformation; 3: total RHD and retracting. Deformation levels 0a and 0b were considered as non-symbiotic. All experiments were performed on at least 3 plants and 5 roots were observed for each plant.

### Statistical Analyses

Statistical analyses were performed on raw data: the number of hairs counted in each level of deformation using the R software package (R Core Team, 2013). A Shapiro-Wilk normality test was performed followed by a non-parametric Kruskal-Wallis multiple comparison test and a pairwise Wilcoxon test. These tests were used to compare the symbiotic response obtained for each treatment.

### Phylogenetic Tree

The strict core genome of 17 *Frankia* strains was determined with the Get_Homologs package (Contreras-Moreira and Vinuesa, 2013). Out of 150,000 amino acid sequences in the *Frankia* pan genome, 420 proteins were identified as orthologs and part of the strict *Frankia* core genome. A concatenated phylogenetic tree was constructed. These concatenations were aligned using Clustal W (Larkin et al., 2007). The distance matrix was computed by Jukes-Cantor method (Jukes and Cantor, 1969). The Neighboring-joining method (Saitou and Nei, 1987) was used to build the phylogeny. The percentage of replicate trees in which the associated taxa clustered together was determined using a bootstrap test (1000 replicates) (Felsenstein, 1985). *Streptomyces coelicolor* was used as an outgroup.

## Results

### CgRHD and CgNINA Activities Are Present in *F. casuarinae* Supernatant Fluid

**FIGURE 2:**
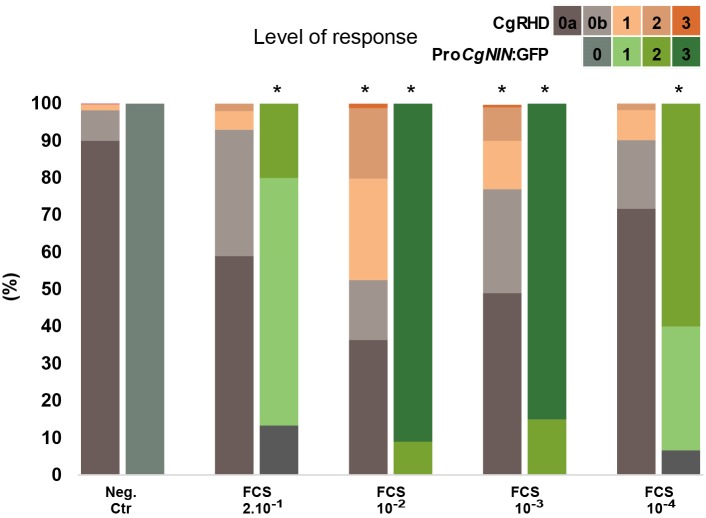
Effect of cell-free *F. casuarinae* supernatant fluids (FCS) on *Casuarina glauca* root hair deformation (CgRHDF bioassay) and the activation of Pro*CgNIN* (CgNINA bioassay). Plants were incubated with *Frankia* culture supernatant fluids (FCS) at the indicated dilutions and the *Frankia* culture medium BAP was used as a negative control. Orange bars represent the proportion of deformed root hairs in short lateral roots 2 days after contact with FCS dilutions. Green bars represent the proportion of plants expressing GFP in short lateral roots at different levels. Asterisks above bars indicate symbiotic responses significantly different from the negative control (*P* < 5%).

Previous studies have shown that factors inducing RHD in *A. glutinosa* (AgRHDF) are present in several *Alnus*-infective *Frankia* strains (Prin and Rougier, 1987; Ghelue et al., 1997; Cérémonie et al., 1999). We recently found that *F. casuarinae* produces an extracellular factor, named CgNINA, which is able to induce the expression of the early symbiotic gene *CgNIN* in small lateral roots (Clavijo et al., 2015; Chabaud et al., 2016). To investigate whether a root hair deforming factor, hereafter named CgRHDF, was also produced by *F. casuarinae*, we incubated wild type *C. glauca* plants with 2 10^-1^, 10^-2^ 10^-3^, and 10^-4^-fold dilutions of *F. casuarinae* supernatant fluids (FCS) and scored the deformation of root hairs situated in small lateral roots. In parallel, transgenic plants containing the Pro*CgNIN:GFP* construct were incubated with the same solutions and the activation of Pro*CgNIN* was recorded. As shown in **Figure 2** and **Supplementary Table S2**, no RHD and no GFP fluorescence were detected in negative control roots treated with diluted BAP medium. RHD and GFP expression were maximal for the 10^-2^ dilution and lower levels of deformation and GFP fluorescence was observed with higher dilutions. The more concentrated dilution had a decreased response for both RHD and GFP fluorescence suggesting that the receptor could be saturated or inhibitory compounds may be associated with the extracts. At the dilutions 10^-1^ and 10^-4^, only the CgNINA bioassay showed a significant difference with the negative control suggesting that the CgNINA bioassay is more sensitive that the one based on CgRHD. The last three dilutions (10^-2^ to 10^-4^) appear to show dose-dependent responses for the CgNINA bioassay that may be used to quantify this factor.

### CgRHDF and CgNINA Share Physio-Chemical Properties

**FIGURE 3:**
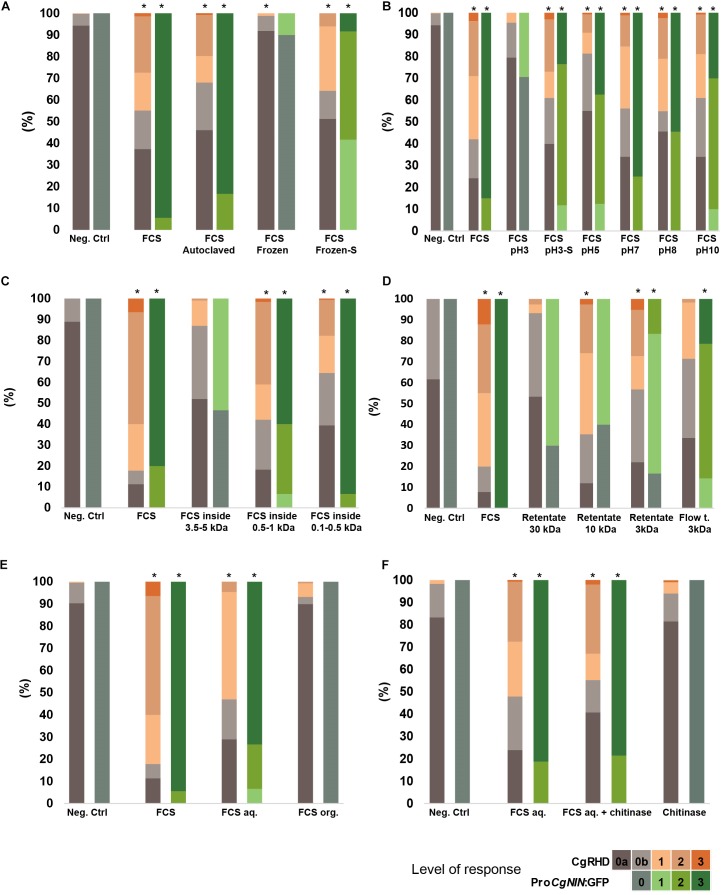
Physico-chemical properties of CgRHDF and CgNINA. Orange bars show the proportion of deformed root hairs in short lateral roots 2 days after contact with FCS submitted to the different treatments. BAP medium diluted 100 times was used as a negative control. Asterisks above bars indicate symbiotic responses significantly different from the negative control (*P* < 5%). **(A)** Temperature sensitivity. High levels of CgRHD and GFP were detected in autoclaved FCS but no significant activity was present in FCS that was previously frozen. Sonication of frozen FCS (Frozen-S) allowed partial recovery of CgRHD and CgNINA activities. **(B)** pH sensitivity: FCS were incubated at different pH for one hour at the indicated pH. Sonication of FCS incubated at pH3 (pH3-S) restored CgRHD and CgNINA levels similar to the untreated control. **(C)** Size estimation using a dialysis tubing. CgRHD and CgNINA activities inside dialysis tubings with the indicated cutoffs was scored **(D)** Size estimation using centrifugal filters. FCS were submitted to successive filtrations using filters with the indicated cut-offs. CgRHD and CgNINA were evaluated on the retentate or the flow through (Flow t.). **(E)** CgRHD and CgNINA activity after 1-butanol extraction. Significant activities were only detected in the aqueous fraction (FCSaq) and not in the organic fraction (FCSorg). **(F)** Sensitivity to Chitinase. FCSaq incubated with chitinase showed similar CgRHD and CgNINA activities compared to untreated FCS.

CgRHDF and CgNINA properties were further compared by using similar treatments to those used to characterize CgNINA (Chabaud et al., 2016). First, the effects of temperature and pH sensitivity were analyzed. As shown in **Figures 3A,B** and **Supplementary Table S2**, CgRHD was not affected by elevated temperatures (autoclaving) or treatment at pH values ranging from 5 to 10. However, cold treatment (freezing) or acidic pH conditions severely decreased CgRHD levels. Sonication of the inactivated fractions (frozen or acid-treated) resulted in a partial recovery of CgRHD activity (**Figures 3A,B** and **Supplementary Table S2**). Similar results were obtained with the CgNINA bioassay. These results suggesting that both factors are thermoresistant and possibly precipitate at low pH or upon freezing but this aggregate can be resuspended using sonication. Both dialysis membranes and centrifugal filters were used to determine the approximate size of CgRHDF and CgNINA. As shown in **Figure 3C** and **Supplementary Table S2**, CgRHDF and CgNINA were detected inside the 100–500 Da and the 500-1000 Da cut-off dialysis tubing, but only a residual activity was found in the 3.5–5 kDa) suggesting that both factors correspond to small molecules with a molecular mass between 1 and 3.5 kDa. Experiments performed using centrifugal filters with 30, 10, and 3 kDa cut-offs yielded similar results for the CgNINA bioassay (**Figure 3D** and **Supplementary Table S2**). However, maximum activity using the CgRHD bioassay was detected in the 3 kDa and 10 kDa retentates and only residual CgRHD was detected in the 3kDa flow though suggesting that both factors correspond to small but distinct molecules. Taken together these experiments suggest that the size of CgNINA is between 1 and 3 kDa while CgRHDF is between 3 and 3.5 kDa. The polarity of the two factors was investigated using a butanol extraction. CgRHDF and CgNINA were only detected in the aqueous phase, indicating that both factors are hydrophilic (**Figure 3E** and **Supplementary Table S2**). Finally, we investigated whether CgRHDF contains a chitin backbone by performing the chitinase digestion experiment described in Chabaud et al. (2016). As shown in **Figure 3F** and **Supplementary Table S2**, the incubation with chitinase had no significant effect on CgRHDF or CgNINA activities. To rule out any inhibitory effect by FCS, we quantified the chitinase activity in the FCS/chitinase solution. As shown in **Supplementary Figure S1**, chitinase activity was not decreased by the addition of FCS to the chitinase solution. We conclude that *F. casuarinae* secretes two factors, CgNINA and CgRHDF, which are possibly two distinct molecules. Both factors sharing similar biochemical properties and correspond to small hydrophilic and thermoresistant molecules lacking a chitin backbone.

### Presence of RHDF and CgNINA in *Frankia* Strains Representative of *Frankia* Diversity

**FIGURE 4:**
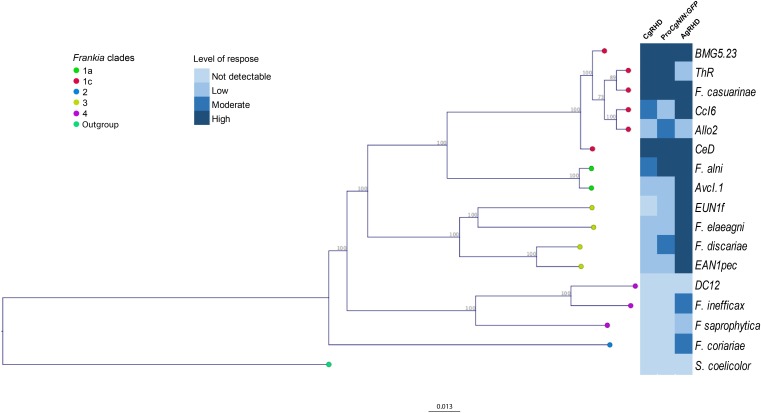
Distribution of CgRHD, CgNINA, and AgRHD activities in *Frankia* strains representative of *Frankia* diversity. A genome-wide phylogenetic tree of 17 *Frankia* strains and *S. coelicolor* was constructed using the concatenated sequences of 420 shared proteins. Distances were computed by the Jukes-Cantor method and the neighbor-joining method was used to build the tree. One thousand bootstrap replications were used to evaluate statistical support for branches. Cell-free supernatant fluids of each strain were tested for their ability to induce RHD and the activation of Pro*CgNIN* on *C. glauca* and RHD on *A. glutinosa*. Levels of response recorded for each strain correspond to the maximum deformation index and the maximum GFP fluorescence level for *C. glauca* and the maximum deformation level for *A. glutinosa*.

We were interested in determining whether other *Frankia* strains had CgNINA and RHDF activities and tested different strains representing *Frankia* diversity. Cell-free supernatant fluids corresponding to 16 *Frankia* strains and another Actinobacteria, S. *coelicolor*, were tested for their capacity to deform *C. glauca* root hairs. As shown in **Figure 4**, **Supplementary Table S3**, and **Supplementary Figure S2**, CgRHD was present in all the *Frankia* belonging to clades I and III. The strongest activities were detected in strains that nodulate the genus *Casuarina* (clade Ic). Remarkably, RHD activity was also detected for several *Frankia* strains unable to form nodules on *Casuarina* such as *F. alni* (Clade Ia, *Alnus*-infective), *Frankia eleagni* and EANIpec (both clade III, *Elaeagnus*-infective strains). No deformation was detected for *Frankia coriariae* (clade II). Supernatant fluids from the atypical strains from clade IV or from the non-*Frankia* actinobacterium *S. coelicolor* did not induce RHD on *Casuarina*. Similar results were obtained when the same supernatant fluids were tested with the NIN bioassay (**Figure 4**, **Supplementary Table S3**, and **Supplementary Figure S2**) except for *F. discariae.*

The strong RHD and NINA activities obtained with the *Alnus*-strain *F. alni* prompted us to investigate whether the same culture supernatant fluids could induce RHD on *A. glutinosa*. As shown in **Figure 4** and **Supplementary Figure S2**, responses observed in *A. glutinosa* were generally similar or stronger compared to the ones recorded in *C. glauca* particularly for strains belonging to group III. Interestingly, strains that were not able to activate ProCgNIN or to deform root hairs of *C. glauca* such as *F. coriariae* and the two atypical strains from group IV, EuI1c and CN3 were able to deform *A. glutinosa* root hairs. To confirm the absence of unintentional contaminations with a compatible strain, *C. glauca, A. glutinosa*, and *O. trinervis* were inoculated with the bacterial pellet obtained while preparing the supernatant fluids described above. As shown in **Supplementary Table S3**, nodules were only obtained with compatible strains, thus demonstrating that responses recorded with incompatible strains are not the result of any contamination; incompatible strains are thus probably able to synthetize CgRHDF and CgNINA.

Surprisingly for *F. discariae*, we detected not only weak levels of RHD in *C. glauca* but also a weak activation of Pro*CgNIN* and a strong deformation of root hairs in *A. glutinosa* (**Figure 4** and **Supplementary Figure S2**). These results are in apparent contradiction with our previous work, in which we could not detect any activation of Pro*CgNIN* in response to *F. discariae* supernatants. However, at that time *F. discariae* cultures were induced with RE from *O. trinervis* instead of *C. glauca* (Chabaud et al., 2016). We therefore repeated the experiments for *F. alni* and *F. discariae* using RE from *A. glutinosa* and *O. trinervis*, respectively. As shown in **Figure 4**, **Supplementary Table S3**, and **Supplementary Figure S2**, incubation of *F. alni* with RE from the host plant (*A. glutinosa*) did not change the response of *C. glauca*, but slightly increased the response of *A. glutinosa*. For *F. discariae*, incubation with exudates from *O. trinervis* reduced the level of Pro*CgNIN* activation compared to the experiment performed with RE from *C. glauca* or without RE and the results obtained were not significantly different from the negative control (**Supplementary Figure S2**). Together, these results showed that *F. discariae* is able to synthetize molecules able to induce CgRHD, AgRHD, and the activation of Pro*CgNIN*. The activation of Pro*CgNIN* was enhanced when *F. discariae* was incubated with RE from *C. glauca* and was possibly below detection limits using the less sensitive equipment described in (Chabaud et al., 2016).

## Discussion

### Presence of CgRHDF and Comparison to CgNINA

In legumes, RHD is one of the earliest visible responses induced upon recognition of rhizobial NFs by the host plant, and the development of a bioassay based on RHD was crucial to identify the chemical nature of NFs (Lerouge et al., 1990). The perception of NFs also provokes significant alterations of gene expression and notably the expression of symbiosis-induced genes such as *MtEnod11* (Journet et al., 2001) and *NIN* (Schauser et al., 1999; Radutoiu et al., 2003). In actinorhizal plants infected intracellularly such as *C. glauca* and *A. glutinosa*, RHD is also one of the first visible responses to *Frankia* inoculation and factors able to induce RHD in *Alnus* (AgRHDF) have been partially purified and characterized (Cérémonie et al., 1999). In *C. glauca*, we have used transgenic plants expressing a Pro*CgNIN:GFP* fusion to characterize CgNINA, a factor present in cell free *F. casuarinae* supernatant fluids able to activate the *CgNIN* promoter in *C. glauca* root hairs. Here we have shown that a CgRHDF is also present in cell-free *F. casuarinae* supernatant fluids. Furthermore, we have shown that CgRHDF and CgNINA share similar physico-chemical properties listed in **Table 1**. Interestingly the experiment performed with centrifugal filters suggests that CgNINA and CgRHDF are both small molecules with slightly different sizes, CgNINA being probably smaller than CgRHDF. This observation is intriguing if CgNINA or CgRHDF are the actinorhizal analogs of rhizobial NFs because NFs are known to induce both RHD and the expression of early nodulins such as NIN. Responses obtained with centrifugal filters were however, less contrasted compared to the other experiments shown here and residual CgRHD activity was still present in the 3kDa flow through. We therefore cannot exclude that CgNINA also possess a small RHD activity and additional experiments are needed to confirm this hypothesis. If CgRHDF and CgNINA can indeed be separated, it would be interesting to know if those molecules are able to induce the high frequency nuclear Ca^2+^ spiking in growing *C. glauca* root hairs as described previously (Chabaud et al., 2016). The ability to induce Ca^2+^ oscillations in response to symbiotic bacteria is a common feature of nodulating species within the nitrogen-fixing clade (Granqvist et al., 2015). Alternatively we hypothesize that CgRHDF and CgNINA are the same molecule but a cofactor or a specific decoration is needed to enhance CgRHD activity without affecting its ability to activate Pro*CgNIN*. The 3 kDa centrifugal filter possibly eliminated decorated molecules with higher mass or cofactors and therefore strong activity was only detected with the CgNINA bioassay.

**Table 1:** Properties of CgRHDF, CgNINA, AgRHDF, and rhizobial NFs.

	CgRHDF	CgNINA	AgRHDF	Rhizobial NF
Induction	Inducible	Inducible	Inducible	Inducible
	(root exudates)	(root exudates)	(root exudates)	(flavonoids)
Size	1–3 kDa	1–3 kDa	1.2–3 kDa	1 kDa
Thermal stability	Thermoresistant	Thermoresistant	Thermoresistant	Thermoresistant
Active concentration	10^-2^–10^-3^ (supernatant)	10^-1^–10^-4^ (supernatant)	10^-3^ 10^-5^ (supernatant)	Down to 10^-12^ M
Hydrophilicity	Hydrophilic	Hydrophilic	Hydrophilic	Amphiphilic
Chitinase action	resistant	resistant	resistant	sensitive


### Comparison With AgRHDF and Rhizobial Nod Factors

CgRHDF and CgNINA also share many characteristics with AgRHDF, the corresponding factor characterized using *A. glutinosa*/*F. alni* (**Table 1**; Ghelue et al., 1997; Cérémonie et al., 1999). However, both factors appear to be structurally different from the rhizobial NFs because unlike NFs they are not found in the organic phase following a butanol extraction and are not sensitive to the endochitinase from *Aeromonas hydrophila* (Cérémonie et al., 1999) or the exochitinase from *S. griseus* (Cérémonie et al., 1999; Chabaud et al., 2016). This difference is in agreement with (1) the lack of *nodA* genes in the sequenced genomes of *F. casuarinae* and *F. alni* (Normand et al., 2007). (2) the absence of chitin oligomers in *F. alni* supernatant fluids (Cérémonie et al., 1999), and (3) the failure of NFs from the broad host-range rhizobia NGR234 to elicit RHD or Ca^2+^ spiking in *A. glutinosa* or *C. glauca* (Cérémonie et al., 1999; Granqvist et al., 2015; Chabaud et al., 2016). The possibility that actinorhizal recognition is mediated by molecules that are not hydrolyzed by tested chitinases is unexpected because downstream components of the NF signaling pathway are conserved not only between actinorhizal and rhizobial symbioses (Svistoonoff et al., 2014; Griesmann et al., 2018), but also between rhizobial and arbuscular-mycorrhizal symbioses where chitin-derived Myc-LCOs and COs play an important role as signaling molecules (Camps et al., 2015). Putative orthologs of NF receptors are present in *C. glauca* and *A. glutinosa* (Hocher et al., 2011). We are currently studying whether these genes play a role in actinorhizal symbioses. Because LysM receptor kinases have been shown to recognize not only chitin-derived molecules but also peptidoglycans and exopolysaccharides (Willmann et al., 2011; Kawaharada et al., 2015), orthologs of genes encoding NF receptors could be involved in the recognition of CgRHDF/NINA or AgRHDF, even if their chemical backbone is not chitin-based.

### Presence of NINA and CgRHDF in Other *Frankia* Strains

In most legumes, NFs allow the specific recognition between the host plant and its symbiotic rhizobia (Masson-Boivin et al., 2009; Oldroyd, 2013). Changes in specific decorations often result in host incompatibility (Dénarié et al., 1996) but NFs from incompatible strains can induce symbiotic responses such as RHD and activation of symbiotic genes when applied at increased concentrations (Roche et al., 1991). These results can be explained by changes of affinity between NFs and the cognate NF receptors able to recognize the chitin backbone and also the modified backbone structure. A misrecognition leads to decreased affinity but this can be compensated by increased amounts of substrate (Dénarié et al., 1996). The symbiotic responses in non-host plants reported here point to a similar mechanism in *C. glauca*: strains from clades I and III possibly synthesize molecules sharing a common molecular backbone that is recognized by *C. glauca* receptors inducing RHD and the activation of Pro*CgNIN* promoter. Optimal recognition is achieved for compatible strains (clade Ic) and some related strains (*F. alni* from clade Ia) but only the backbone would be recognized for more distant strains (clade III). This recognition is not detectable for non-infective strains (clade IV) and the distantly related strain *F. coriariae* suggesting that those strains do not produce sufficient amounts of this recognized backbone under the tested conditions.

### Comparison Between CgRHDF and AgRHDF

Compared to CgRHDF and CgNINA, the distribution of AgRHDF seems less related to phylogeny. Generally, AgRHDF levels were stronger for clade III strains and several strains without any CgRHDF or CgNINA activity (*F. coriariae* and two uninfective strains from clade IV). These differences suggest that the AgRHDF assay detects smaller concentrations of deforming factors or that root hairs of *A. glutinosa* are deformed by a wider range of molecules compared to *C. glauca*. This second hypothesis is in agreement with RHD detected in *Alnus* roots incubated with non-*Frankia* bacteria or fungi (Berry and Torrey, 1983; Knowlton and Dawson, 1983; Prin and Rougier, 1987; Sequerra et al., 1994).

### Impact of Root Exudates

We also found that the nature of RE used to incubate *Frankia* cultures could have an impact on RHDF and CgNINA activities. Unexpectedly lower CgRHDF and CgNINA activities were found when *F. discariae* was cultivated with RE from *O. trinervis* compared to RE from *C. glauca*. In legumes, specific flavonoids secreted by the host plant induce the expression of *nod* genes and the synthesis of NFs (Oldroyd, 2013). RE secreted by the host plant probably also play a role in actinorhizae formation because the incubation with RE induces morphological changes in *Frankia* and accelerate the nodulation process (Gabbarini and Wall, 2008, 2011; Beauchemin et al., 2012). Myricaceae seed extracts also influence *Frankia* growth (Bagnarol et al., 2007). In *Alnus* AgRHDF is reported to be produced either constitutively (McEwan et al., 1992; Ghelue et al., 1997; Cérémonie et al., 1999) or upon induction with RE (Prin and Rougier, 1987). Different plant RE have different effects on *Frankia* physiology. Information about CgRHDF is scarce but flavonoids isolated from *Casuarina* seeds have been shown to induce the production of CgRHDF by the *Casuarina*-infective BR strain (Selim, 1995). Increased CgRHD activity in *F. discariae* incubated with *C. glauca* RE could be due to increased amounts of flavonoids in *Casuarina* RE compared to *O. trinervis.*

## Author Contributions

MC, VH, PP, MS, and SSv conceived and designed the experiments. MC, PP, PF, VH, and AC-M performed the experiments. MC, SSv, PP, PF, and VH analyzed the data. SS and MC statistical analyzed the data. MS, MG, MC, VH, HG, DG, AC-M, SP, AC, MN, ES, CP, and SSv contributed reagents, materials, analysis, and tools. MC, SSv, and LT wrote the paper. All authors read the final version of the manuscript.

## Conflict of Interest Statement

The authors declare that the research was conducted in the absence of any commercial or financial relationships that could be construed as a potential conflict of interest.

## Footnotes

1 https://www.csiro.au/en/Research/Collections/ATSC
