# Supplementary material for: Actinorhizal Signaling Molecules: Frankia Root Hair Deforming Factor Shares Properties With NIN Inducing Factor
Source: Front Plant Sci. 2018 Oct 18;9:1494. doi: 10.3389/fpls.2018.01494 (PMC6201211; doi:10.3389/fpls.2018.01494)
Supplement: Supplementary file 1 [file Image_1.PDF]

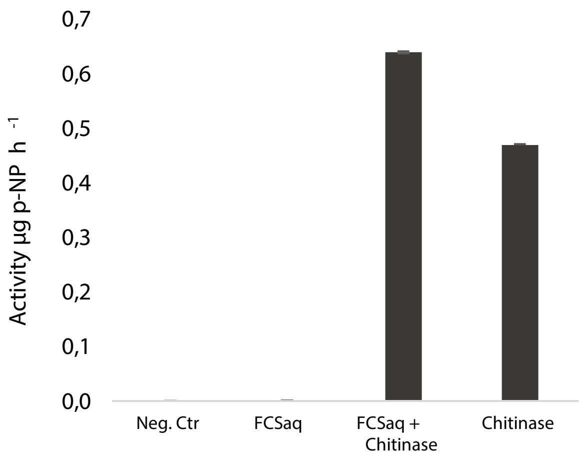

Supplementary figure 1: FCS do not inhibit chitinase activity.
Quantification of chitinase activity using para-nitrophenyl N-acetyl glucosaminide (p-NP-NAG) as substrate. Absorbance at 400 nm was used to determine the release of para nitrophenol (p-NP). Enzyme activity is expressed as µg of liberated p-NP per hour of incubation. Chitinase activity was evaluated in a chitinase solution and in the active aqueous fraction obtained after a 1-butanol extraction (FCSaq) incubated with chitinase. No activity was detected in the *Frankia* culture medium BAP (Neg. Ctr) or in FCSaq without chitinase.
