# Supplementary material for: Actinorhizal Signaling Molecules: Frankia Root Hair Deforming Factor Shares Properties With NIN Inducing Factor
Source: Front Plant Sci. 2018 Oct 18;9:1494. doi: 10.3389/fpls.2018.01494 (PMC6201211; doi:10.3389/fpls.2018.01494)
Supplement: Supplementary file 2 [file Image_2.PDF]

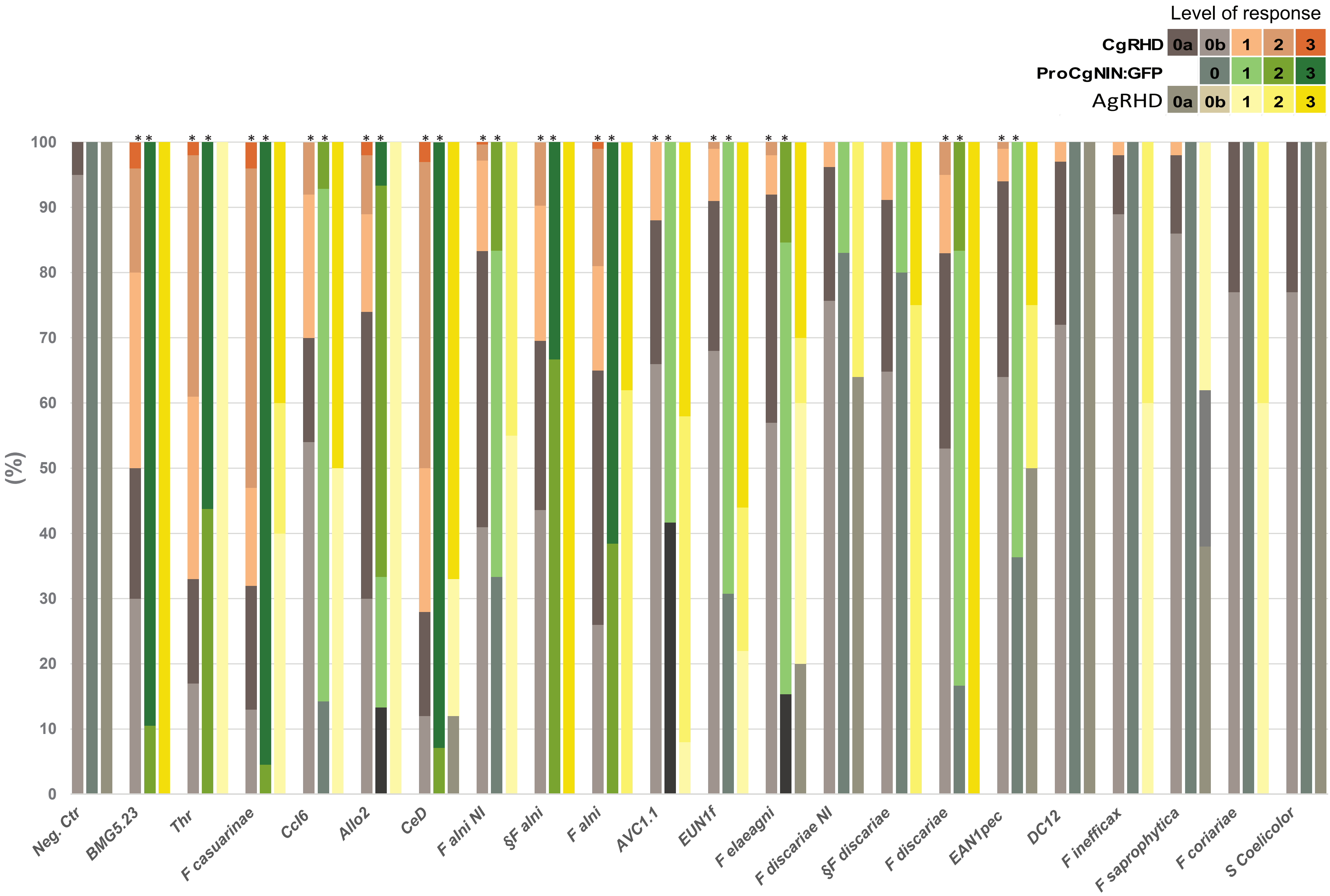

Supplemental figure 2: CgRHD, NINA and AgRHD bioassays performed on supernatant fluids from different strains

Cell-free supernatant fluids of each strain were tested for their ability to induce RHD and the activation of Pro*CgNIN* on *C. glauca* and RHD on *A. glutinosa*. Orange bars represent the proportion of deformed root hairs in short lateral roots of *C. glauca*. Green bars represent the proportion of plants expressing GFP in short lateral roots at different levels. Yellow bars represent the proportion of deformed root hairs in short lateral roots of *A. glutinosa*. § indicate that this strain was cultivated with root exudates from the host plant. NI: strains that were not induced with root exudates. Asterisks above bars indicate symbiotic responses significantly different from the negative control (P<5%). BAP medium diluted 100 times was used as a negative control (Neg. Ctrl).
