# Supplementary material for: Actinorhizal Signaling Molecules: Frankia Root Hair Deforming Factor Shares Properties With NIN Inducing Factor
Source: Front Plant Sci. 2018 Oct 18;9:1494. doi: 10.3389/fpls.2018.01494 (PMC6201211; doi:10.3389/fpls.2018.01494)
Supplement: Supplementary file 3 [file Table_1.PDF]

Supplementary Table 1: List of bacterial strains and the culture conditions used.

Strain name	Group	Host plant	Carbon source in BAP Medium	Temperature	pH	Pre-induction	REFERENCE
Frankia Alni (=ACN14)	Ia	*Alnus crispa*	*Sodium Propionate 5 mM*	*28°C*	*6.7*<br>*6.7*	*A. glutinosa*<br>*C. glauca*	Normand and Lalonde.1982
AVC1.1	Ia	*Alnus viridis*	*Sodium Succinate 20 mM*	*28°C*	*6.7*	*C. glauca*	Baker and Torrey. 1979
Frankia casuarinae (=CcI3)	Ic	*Casuarina cunninghamiana*	*Sodium Propionate 5 mM*	*28°C*	*6.7*	*C. glauca*	Zhang et al. 1984
BMG5.23	Ic	*Casuarina glauca*	*Sodium Propionate 5 mM*	*28°C*	*6.7*	*C. glauca*	Ghodhbane-Gtari et al. 2010
Allo2	Ic	*Alloasuarina verticillata*	*Sodium Propionate 5 mM*	*28°C*	*6.7*	*C. glauca*	Girgis and Schwencke. 1993
CeD	Ic	*Casuarina equisetifolia*	*Sodium Propionate 5 mM*	*28°C*	*6.7*	*C. glauca*	Diem et Dommergues. 1983
CcI6	Ic	*Casuarina cunninghamiana*	*Sodium Propionate 5 mM*	*28°C*	*6.7*	*C. glauca*	Mansour and Moussa. 2005
Thr	Ic	*Casuarina cunninghamiana*	*Sodium Propionate 5 mM*	*28°C*	*6.7*	*C. glauca*	Girgis et al. 1990
Frankia coriariae (=BMG5.1)	II	*Coriaria myrtifolia*	*Sodium Puryvate 5 mM*	*28°C*	*8*	*C. glauca*	*Gtari et al. 2015*
EuN1f	III	*Elaeanus umbellata*	*Fructose 20 mM*	*28°C*	*6.7*	*C. glauca*	Lalonde et al. 1981
Frankia elaeagni (=BMG5.12)	III	*Elaeagnus angustifolia*	*Sodium Propionate 5 mM*	*28°C*	*6.7*	*C. glauca*	Gtari et al. 2004
Frankia discariae (=BCU110501)	III	*Discaria Trinervis*	*Glucose 20 mM*	*28°C*	*6.7*<br>*6.7*	*O. trinervis*<br>*C. glauca*	Chaia. 1998
EAN1pec	III	*Elaeagnus angustifolia*	*Sodium Succinate 20 mM*	*25°C*	*6.7*	*C.glauca*	Normand et al. 2007b
Frankia saprophytica (=CN3)	IV	*Coraria nepalensis*	*Fructose 20 mM*	*28°C*	*6.7*	*C. glauca*	Mirza et al. 1992
Frankia inefficax (=EuI1c)	IV	*Eleagnus umbellata*	*Glucose 20 mM*	*28°C*	*6.7*	*C. glauca*	Beauchemin et al. 2013
DC12	IV	*Datisca canabina L*	*Fructose 20 mM*	*28°C*	*6.7*	*C. glauca*	Hafeez 1983
Streptomyces coelicolor	-	Free living	Sodium Propionate 5 mM	28°C	6.7	C. glauca	Müller 1908

References

• Baker, D and Torrey, J. G. (1980). Characterization of an effective actinorhizal microsymbiont, Frankia Sp. Avc1.1 (Actynomycetales) Can J Microbiol 26 : 1066-1077

• Beauchemin, N. J., Furnholm, T., Lavenus, J., Svistoonoff, S., Doumas, P., Bogusz, D., et al. (2012). Casuarina root exudates alter the physiology, surface properties, and plant infectivity of Frankia sp. strain CcI3. Applied and environmental microbiology 78, 575–580.

• Chaia, E. (1998). Isolation of an effective strain of Frankia from nodules of Discaria trinervis (Rhamnaceae). Plant and Soil 205, 99–102.

• Diem, H. G. and Dommergues, Y. (1983). The isolation of Frankia from nodules of Casuarina. Can J Bot 61 :2822-2825

• Ghodhbane-Gtari, F., Nouioui, I., Chair, M., Boudabous, A., and Gtari, M. (2010). 16S-23S rRNA intergenic spacer region variability in the genus Frankia. Microb. Ecol. 60, 487–495. doi:10.1007/s00248-010-9641-6.

• Girgis, M. G. Z. and Schwencke, J. (1993). Differentiation of Frankia strains by theirs electrophoretic patterns of intercellular esterases and aminopeptidases. J Gen Microbiol. Lett. 234 :349-355

• Girgis, M. G. Z., Ishac, Y. Z., El-Haddad, M., Saleh, E. A., Diem, H. G., and Dommergues, Y. R. (1990). First report on isolation and culture of effective Casuarina compatible strains of Frankia from Egypt, p 156-164.

• Gtari, M., Brusetti, L., Skander, G., Mora, D., Boudabous, A., and Daffonchio, D. (2004). Isolation of Elaeagnus-compatible Frankia from soils collected in Tunisia. FEMS Microbiol. Lett. 234, 349–355. doi:10.1016/j.femsle.2004.04.001.

• Gtari, M., Ghodhbane-Gtari, F., Nouioui, I., Ktari, A., Hezbri, K., Mimouni, W., et al. (2015). Cultivating the uncultured: growing the recalcitrant cluster-2 Frankia strains. Scientific reports 5.

• Hafeez, F. (1983). Nitrogen fixation and nodulation in Datisca cannabina L. and Alnus nitida Endl. PhD thesis. Quaid-e-Azam University, Islamabad, Pakistan

• Mansour, S. R., and Moussa, L. A. A. (2005). Role of Gamma-radiation on spore germination and infectivity of Frankia strains CeI523 and CcI6 isolated from Egyptian Casuarina. Isotope Rad. Res 37, 1023–1038.

• Mirza, M. S., Hahn, D., Akkermans, A. D. L. (1992). Isolation and characterization of Frankia strains from Coriaria nepalensis. System. Appl. Microbiol. 15 :289-295

• Müller R (1908). Eine Diphteridee und eine Streptothrix mit gleichem blauen Farbstoff, sowie Untersuchungeri uber Streptothrix-Arten im Allgemeinen. 46, 195–212.

• Normand, P., and Lalonde, M. (1982). Evaluation of Frankia strains isolated from provenances of two Alnus species. Canadian Journal of Microbiology 28, 1133–1142.

• Normand, P., Lapierre, P., Tisa, L. S., Gogarten, J. P., Alloisio, N., Bagnarol, E., et al. (2007). Genome characteristics of facultatively symbiotic Frankia sp. strains reflect host range and host plant biogeography. Genome research 17, 7–15.

• Zhang, Z., Lopez, M. F., Torrey, J. G. (1984) A comparison of cultural characteristics and infectivity of Frankia isolates from root nodules of Casuarina species. In: Frankia symbioses. Springer, pp 79–90
