# Supplementary material for: Actinorhizal Signaling Molecules: Frankia Root Hair Deforming Factor Shares Properties With NIN Inducing Factor
Source: Front Plant Sci. 2018 Oct 18;9:1494. doi: 10.3389/fpls.2018.01494 (PMC6201211; doi:10.3389/fpls.2018.01494)
Supplement: Supplementary file 4 [file Table_2.PDF]

Supplementary Table 2: CgRHD and NINA bioassays performed on *Frankia casuarinae* supernatant fluids (FCS) submitted to different treatments.

Bioassay	CgRHD							NINA					
**Treatments**	Maximum	0a	0b	1	2	3	Statistical groups	Maximum	0	1	2	3	Statistical groups
**Effect of FCS dilution**													
Neg. Ctr	1	2475	156	17	0	0	*a*	0	22	0	0	0	*a*
FCS 1/20	2	2378	204	204	0	0	*ac*	3	0	0	4	22	*b*
FCS 1/100	4	1142	695	749	432	63	*b*	3	2	10	3	0	*b*
FCS 1/1000	3	1946	707	309	224	7	*bc*	3	0	0	2	13	*b*
FCS 1/10000	2	2459	556	390	31	2	*ac*	2	1	5	9	0	*b*
**Effect of temperature**													
Neg. Ctr	1	1120	30	0	0	0	*a*	0	22	0	0	0	*a*
FCS	4	990	260	175	300	40	*b*	3	0	0	0	17	*b*
FCS Autoclaved	4	1197	814	530	581	13	*b*	3	0	0	2	10	*b*
FCS Frozen	1	2592	378	26	0	0	*c*	1	9	1	0	0	*ac*
FCS Frozen sonicated	2	1728	632	292	184	5	*b*	2	0	5	6	1	*b*
**Effect of pH**													
Neg. Ctr	1	1488	276	8	0	0	*a*	0	13	0	0	0	*a*
FCS	4	1150	395	750	381	44	*b*	3	0	0	3	17	*bc*
FCS pH3	1	2455	367	148	0	0	*a*	1	12	5	0	0	*ac*
FCS pH3 sonicated	4	1550	974	812	240	1	*b*	3	0	1	9	5	*bc*
FCS pH5	3	2114	351	331	333	19	*b*	3	0	1	4	7	*bc*
FCS pH7	3	1299	413	252	388	10	*b*	3	0	0	3	9	*bc*
FCS pH8	3	1526	248	252	350	9	*b*	3	0	0	5	5	*b*
FCS pH10	4	1937	200	445	449	16	*b*	3	0	1	5	3	*b*
**Dialysis experiments**													
Neg. Ctr	1	1826	348	19	0	0	*a*	0	12	0	0	0	*a*
FCS	4	830	268	792	457	70	*bc*	3	0	0	3	12	*b*
3,5 – 5 kDa	2	1644	748	221	0	0	*ab*	1	3	8	0	0	*ab*
0,5 – 1 kDa	4	558	403	280	456	16	*cd*	3	0	1	5	9	*b*
0.1 – 0.5 kDa	4	1590	470	805	623	25	*d*	3	0	0	1	14	*b*
**Centrifugal filters**													
Neg. Ctr	1	1096	251	17	0	0	*b*	0	9	0	0	0	*a*
FCS	4	975	565	235	500	45	*c*	3	0	0	0	9	*b*
Retentate 30 KDa	2	970	660	68	9	0	*bc*	1	3	5	0	0	*ab*
Retentate 10 KDa	4	660	900	1150	430	110	*a*	1	4	6	0	0	*ab*
Retentate 3KDa	4	720	1330	730	815	165	*a*	2	2	6	2	0	*b*
Flow through 3 KDa	3	1260	1540	1100	60	0	*ac*	3	0	2	9	3	*b*
**Butanol extraction**													
Neg. Ctr	1	859	200	24	0	0	*a*	0	13	0	0	0	*a*
FCS	4	1176	331	318	247	43	*b*	3	0	0	1	17	*b*
FCSaq	3	2274	345	391	92	17	*b*	3	0	1	3	11	*b*
FCSorg	1	2780	440	7	0	0	*a*	0	13	0	0	0	*a*
**Effect of chitinase**													
Neg. Ctr	1	1206	462	13	0	0	*a*	0	17	0	0	0	*a*
FCSaq + Chitinase	4	750	441	638	864	82	*b*	3	0	0	3	13	*b*
FCSaq	4	879	448	564	576	25	*b*	3	0	0	3	11	*b*
Chitinase	1	2861	453	109	0	0	*a*	0	9	0	0	0	*a*

CgRHD: Root Hair Deformation bioassay in *C. glauca*. The total number of root hairs showing the corresponding deformation level is indicated for each treatment. Levels 0a and 0b were considered non symbiotic. Different letters indicate significantly different symbiotic responses (P<5%). **NINA bioassay:** the activation of Pro*CgNIN:GFP* was determined using the following fluorescence scale: 0: no detectable fluorescence; 1: weak fluorescence; 2: intermediate fluorescence; 3: strong fluorescence. Level 0 was considered non symbiotic. Different letters indicate significantly different symbiotic responses (P<5%). Neg. Ctr: BAP medium diluted 100 times used as a negative control. FCSaq: aqueous fraction; FCSorg: organic fraction
