# Supplementary material for: Actinorhizal Signaling Molecules: Frankia Root Hair Deforming Factor Shares Properties With NIN Inducing Factor
Source: Front Plant Sci. 2018 Oct 18;9:1494. doi: 10.3389/fpls.2018.01494 (PMC6201211; doi:10.3389/fpls.2018.01494)
Supplement: Supplementary file 5 [file Table_3.PDF]

Supplementary Table 3: CgRHD and NINA bioassays performed on supernatant fluids from different strains

Strains	CgRHD bioassay						NINA bioassay					Nodulation test
	0a	0b	1	2	3	Statistical groups	0	1	2	3	Statistical groups	
Negative Control (BAP)	3327	318	35	0	0	fgh	22	0	0	0	i	-
BMG5.23	831	614	486	524	64	ab	0	0	2	17	ab	+Cg
ThR	*413*	416	379	825	17	ab	0	0	7	9	a	+Cg
Frankia casuarinae	819	542	552	1000	67	a	0	0	1	21	abc	+Cg. -Ag. -Ot
CcI6	*600*	720	375	18	0	cd	2	11	1	0	cdef	+Cg
Allo2	683	1353	487	90	0	cd	2	3	9	1	abcd	+Cg
CeD	*134*	349	332	933	14	ab	0	0	1	13	abcd	+Cg
§*Frankia alni*	*1133*	1226	330	204	0	cd	0	3	7	4	abcd	-Cg. +Ag. -Ot
Frankia alni	*1327*	1193	396	227	2	bc	0	0	5	8	bcde	-Cg. +Ag. -Ot
Frankia alni non induced	*666*	485	193	22	2	de	0	5	4	0	cdefg	-Cg. +Ag. -Ot
AvcI.1	*1531*	527	255	20	0	cde	5	7	0	0	gh	-Cg
EUN1f	*1810*	855	199	2	0	de	4	9	0	0	fg	-Cg
Frankia elaeagni	*1080*	1046	203	7	0	de	2	9	2	0	defg	-Cg
§*Frankia discariae*	*1322*	673	383	0	0	de	8	4	0	0	hi	-Cg. -Ag. +Ot
Frankia discariae	*1487*	705	291	57	2	cd	2	8	2	0	efg	-Cg. -Ag. +Ot
Frankia discariae non induced	*610*	217	0	0	0	h	7	2	0	0	hi	-Cg. -Ag. +Ot
EAN1pec	*1436*	1025	192	7	1	de	4	7	0	0	gh	-Cg
DC12	*2133*	646	54	3	0	fgh	10	0	0	0	i	nd
F inefficax	*2133*	263	18	0	0	gh	14	0	0	0	i	-Cg
F saprophytica	*1628*	389	65	0	0	fg	12	0	0	0	i	-Cg
Frankia coriariae	*566*	161	0	0	0	h	9	0	0	0	i	-Cg
Streptomyces coelicolor	1903	885	11	0	0	gh	9	0	0	0	i	nd

For the Root Hair Deformation bioassay in *C. glauca*, the total number of root hairs showing the corresponding deformation level is indicated for each strain. Levels 0a and 0b were considered non symbiotic. Different letters indicate significantly different symbiotic responses (P<5%). For the NINA bioassay the activation of Pro*CgNIN:GFP* was determined using the following fluorescence scale: 0: no detectable fluorescence; 1: weak fluorescence; 2: intermediate fluorescence; 3: strong fluorescence. Level 0 was considered non symbiotic. Different letters indicate significantly different symbiotic responses (P<5%). Neg. Ctr: BAP medium diluted 100 times used as a negative control. Nodulation tests were performed on *C. glauca* (Cg), *A. glutinosa* (Ag) and *O. trinervis* (Ot) using the bacterial pellets obtained while preparing the supernatant fluids. (+) indicate the presence of nodules (-) indicate the absence of nodules. nd: not determined.
